# A multidisciplinary team care approach improves outcomes in high-risk pediatric neuroblastoma patients

**DOI:** 10.18632/oncotarget.13874

**Published:** 2016-12-10

**Authors:** Hsiu-Hao Chang, Yen-Lin Liu, Meng-Yao Lu, Shiann-Tarng Jou, Yung-Li Yang, Dong-Tsamn Lin, Kai-Hsin Lin, Kai-Yuan Tzen, Ruoh-Fang Yen, Ching-Chu Lu, Chia-Ju Liu, Steven Shinn-Forng Peng, Yung-Ming Jeng, Shiu-Feng Huang, Hsinyu Lee, Hsueh-Fen Juan, Min-Chuan Huang, Yung-Feng Liao, Ya-Ling Lee, Wen-Ming Hsu

**Affiliations:** ^1^ Department of Pediatrics, National Taiwan University Hospital and National Taiwan University College of Medicine, Taipei, Taiwan; ^2^ Department of Pediatrics, Taipei Medical University Hospital, Taipei, Taiwan; ^3^ Department of Laboratory Medicine, National Taiwan University Hospital and National Taiwan University College of Medicine, Taipei, Taiwan; ^4^ Department of Nuclear Medicine, National Taiwan University Hospital and National Taiwan University College of Medicine, Taipei, Taiwan; ^5^ Department of Medical Imaging, National Taiwan University Hospital and National Taiwan University College of Medicine, Taipei, Taiwan; ^6^ Department of Pathology, National Taiwan University Hospital and National Taiwan University College of Medicine, Taipei, Taiwan; ^7^ Division of Molecular and Genomic Medicine, National Health Research Institutes, Miaoli County, Taiwan; ^8^ Department of Life Science, National Taiwan University, Taipei, Taiwan; ^9^ Institutes of Zoology, National Taiwan University, Taipei, Taiwan; ^10^ Institutes of Molecular and Cellular Biology, National Taiwan University, Taipei, Taiwan; ^11^ Graduate Institute of Anatomy and Cell Biology, National Taiwan University College of Medicine, Taipei, Taiwan; ^12^ Institute of Cellular and Organismic Biology, Academia Sinica, Taipei, Taiwan; ^13^ Department of Nursing, National Taiwan University College of Medicine, Taipei, Taiwan; ^14^ Department of Nursing, Central Taiwan University of Science and Technology, Taichung, Taiwan; ^15^ Department of Surgery, National Taiwan University Hospital and National Taiwan University College of Medicine, Taipei, Taiwan

**Keywords:** chemotherapy, high-risk, multidisciplinary team care, neuroblastoma, outcomes

## Abstract

We assessed the impact of a multidisciplinary team care program on treatment outcomes in neuroblastoma patients. Newly diagnosed neuroblastoma patients received treatment under the Taiwan Pediatric Oncology Group (TPOG) N2002 protocol at the National Taiwan University Hospital beginning in 2002. A multidisciplinary team care approach that included nurse-led case management for patients treated under this protocol began in January 2010. Fifty-eight neuroblastoma patients, including 29 treated between 2002 and 2009 (Group 1) and 29 treated between 2010 and 2014 (Group 2), were enrolled in the study. The 5-year overall survival (OS) and event-free survival (EFS) rates for all 58 patients were 59% and 54.7%, respectively. Group 2 patients, who were treated after implementation of the multidisciplinary team care program, had better 3-year EFS (*P* = 0.046), but not OS (*P* = 0.16), rates than Group 1 patients. In a multivariate analysis, implementation of the multidisciplinary team approach was the only significant independent prognostic factor for neuroblastoma patients. In further subgroup analyses, the multidisciplinary team approach improved EFS, but not OS, in patients with stage 4 disease, those in the high-risk group, and those with non-MYCN amplified tumors. These data indicate a multidisciplinary team care approach improved survival outcomes in high-risk neuroblastoma patients. However, further investigation will be required to evaluate the long-term effects of this approach over longer follow-up periods.

## INTRODUCTION

Neuroblastoma is a childhood tumor that is derived from neural crest sympathoadrenal lineage progenitor cells. It can develop anywhere in the sympathetic nervous system, but occurs most often in the adrenal gland [1]. Neuroblastoma is the most common extra-cranial solid tumor in children and the most frequently diagnosed malignancy in infants. It is exceedingly rare in adults; more than 96% of neuroblastoma patients are diagnosed when they are less than 10 years old [2].

The clinical course of neuroblastoma is heterogeneous [1], and neuroblastoma patients can be classified into different risk groups based on relevant biological or prognostic factors [3]. The clinical presentations of neuroblastoma can be distinguished into three distinct patterns before treatment according to International Neuroblastoma Risk Group Staging System: (1) locoregional disease with the absence or presence of image-defined risk factors, referred to as L1 or L2 disease, respectively; (2) distant metastatic disease, referred to as M disease; and (3) metastatic disease in children younger than 18 months with metastases confined to skin, liver, and/or bone marrow, referred to as MS disease [4]. Despite recent improvements in treatment, the prognosis remains extremely poor for metastatic neuroblastoma patients who are diagnosed at less than 18 months of age.

The current treatment for neuroblastoma is risk-adapted multimodal therapy, which may include combinations of chemotherapy, radiotherapy, high-dose autologous peripheral blood stem cell (PBSC) transplantation, and surgery [3]. The successful application of such multimodal therapeutic protocols involves cooperation and effective communication among healthcare professionals from a variety of different disciplines. This multidisciplinary team care approach is now recommended for many types of cancer [5, 6] and patients whose treatment is managed by such a team have better survival outcomes [7]. However, few studies have examined the benefits of a multidisciplinary team care approach in pediatric cancer patients [8, 9].

In our hospital, we began treating neuroblastoma patients using the Taiwan Pediatric Oncology Group (TPOG) N2002 protocol, a risk-adapted multimodal therapy, in 2002. This protocol was adapted from the previously described N7 [10], COG-A3961 [11], and COG-3973 [12] protocols, and was designed and supported by the TPOG and Taiwan Childhood Cancer Foundation. We later introduced a comprehensive, multidisciplinary team care program for neuroblastoma patients treated under the TPOG N2002 protocol in January 2010. The aim of the present study was to assess the impact of the multidisciplinary team care program on survival outcomes in neuroblastoma patients as indicated by event-free survival (EFS) and overall survival (OS).

## RESULTS

### Patient characteristics

**Figure 1:**
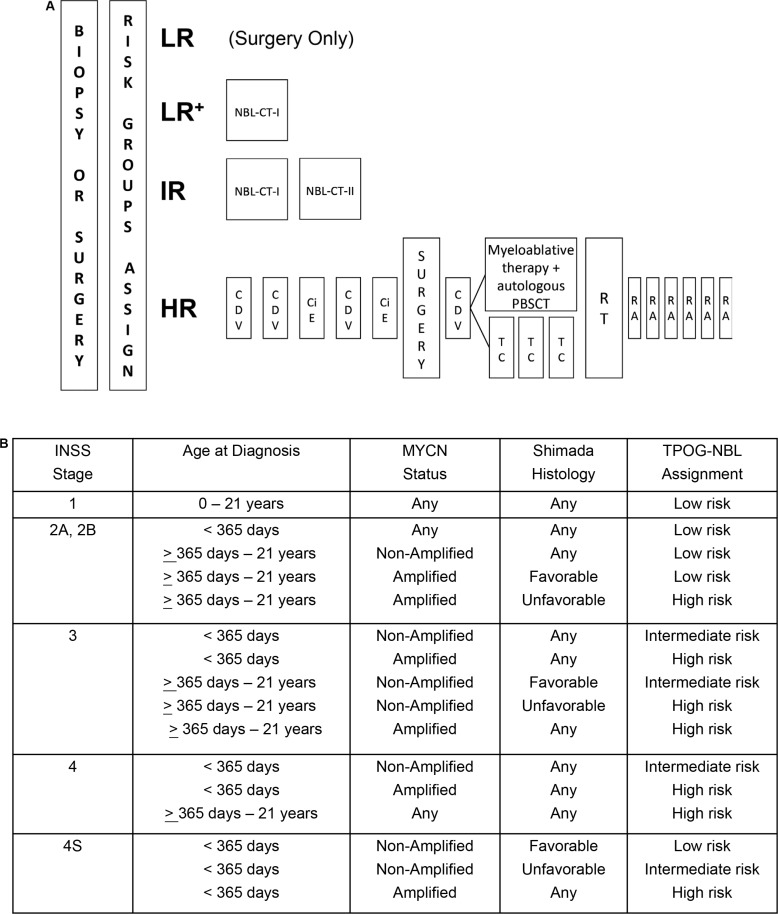
Treatment schematic and (**B**) risk group assignments for the Taiwan Pediatric Oncology Group N2002 protocol. Chemotherapy regimen details are provided in the Materials and Methods. (LR=low-risk group, LR^+^= low-risk group treated with NBL-CT-I chemotherapy regimen, IR=intermediate-risk group, HR=high-risk group, RT=radiotherapy, RA=13-*cis*-retinoid acid therapy).

Between January 2002 and December 2014, 64 new neuroblastoma cases were diagnosed at the National Taiwan University Hospital, Taipei, Taiwan. Among these, 58 patients (90.6%) were treated under the TPOG N2002 protocol (Figure 1, Table 1) and were enrolled for analysis. The remaining 6 patients (9.4%) were excluded because they either were treated under a different protocol or refused treatment altogether. The mean age of the 58 patients was 2.7 years (range, 0.04–15.3 years), and the median follow-up time for patients still alive without an event was 3.6 years (range, 0.6–7.6 years). A summary of the demographic information and clinical characteristics of these patients is provided in Table 2.

**Table 1:** Chemotherapy regimen for low- (NBL-CT-I) and intermediate-risk (NBL-CT-I plus NBL-CT-II) neuroblastoma patients in the Taiwan Pediatric Oncology Group N2002 protocol

Treatment protocol	Cycle (day)	Drug	Dose (mg/m^2^/day)	Dose (mg/kg/day)^†^
NBL-CT-I	1 (1)	Carboplatin	560	18
	1 (1–3)	Etoposide	120	4
	2 (1)	Carboplatin	560	18
	2 (1)	Cyclophosphamide	1,000	33
	2 (1)	Doxorubicin	30	1
	3 (1)	Cyclophosphamide	1,000	3
	3 (1-3)	Etoposide	120	4
	4 (1)	Carboplatin	560	18
	4 (1-3)	Etoposide	120	4
	4 (1)	Doxorubicin	30	1
NBL-CT-II	5 (1)	Cyclophosphamide	1,000	33
	5 (1–3)	Etoposide	120	4
	6 (1)	Carboplatin	560	18
	6 (1)	Cyclophosphamide	1,000	3
	6 (1)	Doxorubicin	30	1
	7 (1)	Carboplatin	560	18
	7 (1–3)	Etoposide	120	4
	8 (1)	Cyclophosphamide	1,000	3
	8 (1)	Doxorubicin	30	1

† Pediatric patients < 12 kg or < 12 months of age.

**Table 2:** Clinicopathological characteristics of pediatric patients (*n* = 58) with newly diagnosed neuroblastoma who were eligible for analysis

Clinicopathological characteristic	Patients (*n* = 58)
Age at diagnosis, years	
Mean (range)	2.7 (0.05–15.3)
≤ 1.5 years, *n* (%)	24 (41.4)
>1.5 years, *n* (%)	34 (58.6)
Sex, *n* (%)	
Male	38 (65.5)
Female	20 (34.5)
INSS stage, *n* (%)	
1	6 (10.3)
2	2 (3.5)
3	12 (20.7)
4	32 (55.2)
4S	6 (10.3)
Primary tumor site, *n* (%)	
Adrenal	46 (79.3)
Extra-adrenal	12 (20.7)
*MYCN*, *n* (%)	
Amplified	14 (24.1)
Non-amplified	44 (75.9)
TPOG risk group, *n* (%)	
Low	10 (17.2)
Intermediate	7 (12.1)
High	41 (70.7)

INSS = International Neuroblastoma Staging System, TPOG = Taiwan Pediatric Oncology Group.

### Outcomes of the TPOG N2002 treatment protocol

Ten patients (17.2%) were assigned to the low-risk group, 7 patients (12.1%) were assigned to the intermediate-risk group, and 41 patients (70.7%) were assigned to the high-risk group prior to treatment under the TPOG N2002 protocol (Figure 1B). The 5-year OS and EFS rates (± standard error) for the 58 enrolled patients were 59 ± 7.8% and 54.7 ± 7%, respectively.

Of the 10 patients in the low-risk group, the 3 patients treated with surgery alone and without chemotherapy (LR in Figure 1A) did not have any tumor recurrences. The remaining 7 low-risk patients underwent an initial tumor biopsy (*n* = 1) or excisional biopsy (*n* = 6) procedure, followed by chemotherapy (LR^+^ in Figure 1A; NBL-CT-I, Table 1). For the patient underwent an initial tumor biopsy, not excisional biopsy, a second operation was performed following chemotherapy, and radiotherapy was used to treat the residual tumor. One (10%) low-risk group patient experienced tumor relapse. He was 8 years old at initial diagnosis and his tumor was localized to the left supra-renal area with regional lymph node involvement. The tumor was classified as stage INSS 2B after initial excisional biopsy and had unfavorable histology. *MYCN* was not amplified in the tumor. The patient received NBL-CT-I chemotherapy followed by local radiotherapy. Tumor relapse occurred in his right scapula after 1 year of treatment and was rescued successfully with high-dose chemotherapy and an autologous PBSC transplantation.

All 7 patients in the intermediate-risk group underwent an initial tumor biopsy (*n* = 2) or excisional biopsy (*n* = 5) procedure, followed by chemotherapy (NBL-CT-I plus NBL-CT-II; Table 1). In 2 patients, a second operation was performed following chemotherapy, and radiotherapy was used to treat the residual tumor. One patient (14.3%) experienced tumor progression after 3 months of treatment and was moved to the high-risk protocol. This patient was one month old at the time of diagnosis. His tumor was initially located at the right retroperitoneum and metastasized to his left thigh. The tumor was classified as stage INSS 4 and had unfavorable histology. *MYCN* was not amplified in the tumor. Array comparative genomic hybridization analysis of tumor samples showed partial 1p and 11q losses and a partial 17q gain. This tumor did not respond well to the high-risk protocol treatment, and the patient was under hospice care at the time of last follow-up.

Of the 41 patients in the high-risk group, 26 patients (63.4%) received autologous PBSC transplantation as a consolidation therapy. However, 15 patients (36.6%) received maintenance therapy instead, which consisted of 3 cycles of cyclophosphamide in combination with topotecan (TC in Figure 1A). Among these 41 patients, 38 were eligible for tumor resection surgery; gross total resection of tumor was achieved in 25 (65.6%) of these patients.

**Figure 2:**
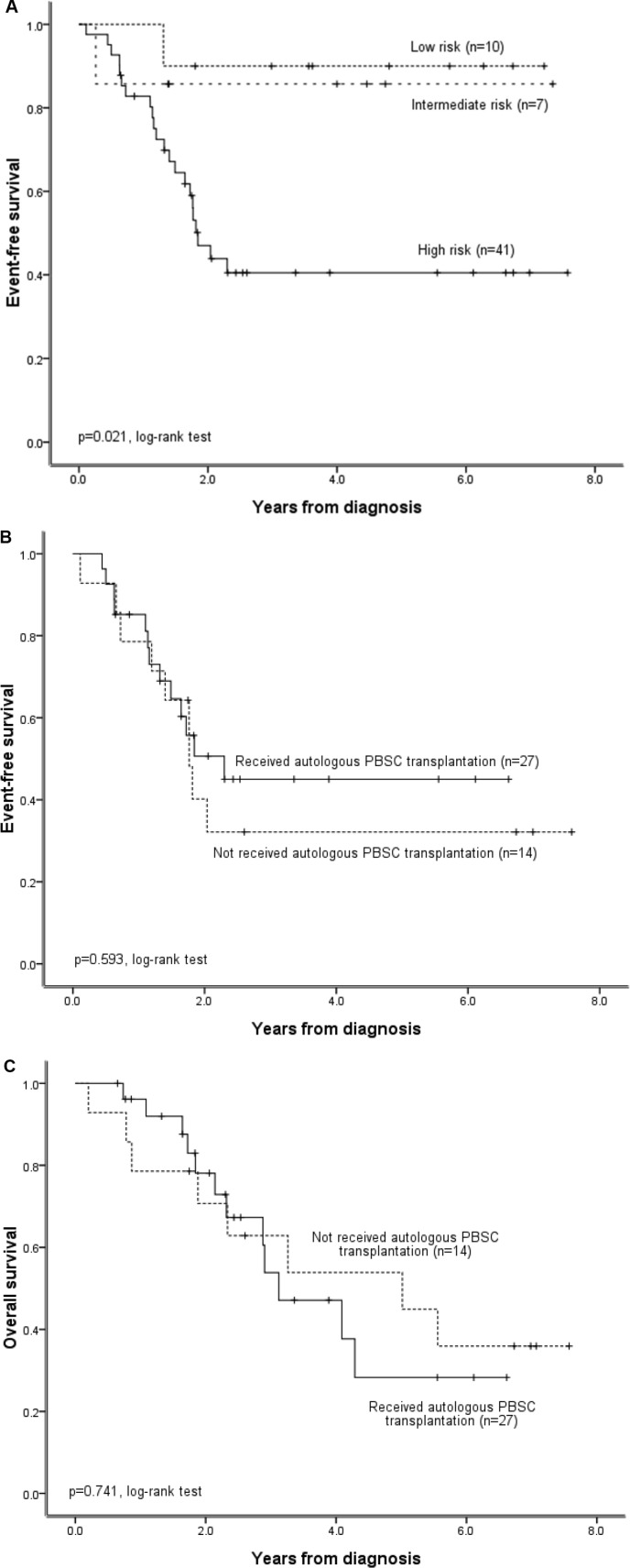
Survival outcomes for neuroblastoma patients (n = 58) treated under the Taiwan Pediatric Oncology Group N2002 protocol (**A**) Probability of EFS in patients in the low-, intermediate-, and high-risk groups. (**B**) Probability of EFS and (**C**) OS in high-risk patients who did or did not receive an autologous PBSC transplantation.

Survival outcomes differed among patients in the low-, intermediate-, and high-risk groups. The 5-year OS rates for low-, intermediate-, and high-risk patients were 100%, 100%, and 41.2 ± 9.5%, respectively (*P* = 0.002 using a log-rank test). The 5-year EFS rates for low-, intermediate-, and high-risk patients were 90 ± 9.5%, 85.7 ± 13.2%, and 40.5 ± 8.3%, respectively (*P* = 0.02 using a log-rank test; Figure 2A). Thus, patients in the high-risk group had poorer survival outcomes than patients in the low- or intermediate-risk groups. However, survival outcomes did not differ in high-risk group patients who received an autologous PBSC transplantation after induction chemotherapy compared to those who did not receive transplantation (*P* > 0.05 using a log-rank test; Figure 2B, 2C).

### Impact of multidisciplinary team care on survival

To evaluate the effects of our multidisciplinary team approach to neuroblastoma care on OS and EFS, we stratified our 58 patients into 2 groups depending on whether they were diagnosed before (Group 1, 2002–2009) or after (Group 2, 2010–2014) the implementation of this approach in January 2010. Groups 1 and 2 contained 29 patients each. Clinical characteristic distributions were comparable between the two groups, except for the number of high-risk patients receiving an autologous PBSC transplantation (Table 3). The impact of selected clinical and biological variables on OS and EFS was analyzed in these patients. An age at diagnosis of > 1.5 years, advanced clinical stage (Stage 3/4) and high-risk status were identified in the univariate analysis as adverse prognostic factors for both 3-year OS and EFS. Patients with *MYCN*-amplified tumors tended to exhibit poorer survival outcomes than patients with non-*MYCN*-amplified tumors, but this difference did not reach statistical significance. Implementation of the multidisciplinary team approach was a favorable prognostic factor for 3-year EFS, but not OS, in the univariate analysis (Table 4). In the multivariate analysis, the implementation of the multidisciplinary team approach was the only independent favorable prognostic factor for EFS, but not OS, in the neuroblastoma patients (Group 2 *vs*. Group 1; Table 5).

**Table 3:** Comparison of patient clinicopathological characteristics before (Group 1; *n* = 29) and after (Group; *n* = 29) implementation of our multidisciplinary team care program

Clinicopathological characteristic	Group 1 (2002-2009), *n* = 29	Group 2 (2010-2014), *n* =29	*P*-value
Age at diagnosis			
≤ 1.5 years	11 (37.9)	13 (44.8)	
> 1.5 years	18 (62.1)	16 (55.2)	0.59
Sex, *n* (%)			
Male	18 (62.1)	20 (69.0)	
Female	11 (37.9)	9 (31.0)	0.58
INSS stage, *n* (%)			
1, 2, 4S	7 (24.1)	7 (24.1)	
3, 4	22 (75.9)	22 (75.9)	> 0 .99
Primary tumor site, *n* (%)			
Adrenal	21 (72.4)	25 (86.2)	
Extra-adrenal	8 (27.6)	4 (13.8)	0.20
*MYCN*, *n* (%)			
Amplified	8 (27.6)	6 (20.7)	
Non-amplified	21 (72.4)	23 (79.3)	0.54
TPOG risk group, *n* (%)			
Low	4 (13.8)	6 (20.7)	
Intermediate	2 (6.9)	5 (17.2)	
High	23 (79.3)	18 (62.1)	0.32
Autologous PBSC transplant^†^, *n* (%)			
Received	11 (47.8)	16 (88.9)	
Not received	12 (52.2)	2 (11.1)	0.006*

^†^High-risk patients only.

*P < 0.05.

INSS = International Neuroblastoma Staging System, PBSC = peripheral blood stem cell, TPOG = Taiwan Pediatric Oncology Group.

**Table 4:** Univariate analysis of prognostic factors in pediatric patients (*n* = 58) with newly diagnosed neuroblastoma

Prognostic factor	Patients (*n* = 58)	3-year EFS, % (SE)	*P*-value	3-year OS, % (SE)	*P*-value
Age at diagnosis					
≤ 1.5 years	24	78.7 (8.5)		91.5 (5.8)	
> 1.5 years	34	35.6 (9.2)	0.012*	56.3 (9.7)	0.001*
INSS stage					
1, 2, 4S	14	85.7 (9.4)		92.9 (6.9)	
3, 4	44	43.0 (8.3)	0.016*	61.4 (8.7)	0.006*
Primary tumor site					
Adrenal	46	55.5 (7.8)		71.5 (7.4)	
Extra-adrenal	12	53.0 (15.5)	0.708	70.0 (14.5)	0.479
MYCN					
Amplified	14	39.2 (14.0)		49 (15.5)	
Non-amplified	44	59.3 (8.0)	0.147	77.9 (7)	0.076
TPOG risk group					
Low	10	90.0 (9.5)		100	
Intermediate	7	85.7 (13.2)		100	
High	41	40.5 (8.3)	0.007*	58.1 (8.85)	0.001*
Implementation of MTC program					
Group 1 (2002-2009)	29	41.4 (9.1)		65.5 (8.8)	
Group 2 (2010-2014)	29	72.8 (9.0)	0.046*	79.9 (9.2)	0.162

* *P* < 0.05.

EFS = event-free survival, INSS = International Neuroblastoma Staging System, MTC = multidisciplinary team care, OS = overall survival, SE = standard error, TPOG = Taiwan Pediatric Oncology Group.

**Table 5:** Multivariate analysis of prognostic factors in pediatric patients (*n* = 58) with newly diagnosed neuroblastoma

Prognostic factor	Patients (*n* = 58)	EFS		OS	
		HR (95% CI)	*P*–value	HR (95% CI)	*P*–value
Age at diagnosis					
≤ 1.5 years	24	0.558 (0.172–1.809)	0.331	0.222 (0.400–1.232)	0.085
> 1.5 years	34	1		1	
INSS stage					
1, 2, 4S^†^	14	1		1	
3, 4^‡^	44	4.023 (0.698–23.172)	0.119	3.979 (0.364–43.532)	0.258
Primary tumor site					
Adrenal	46	2.236 (0.771–6.481)	0.139	2.573 (0.791–8.374)	0.116
Extra-adrenal	12	1		1	
MYCN					
Amplified	14	1.033 (0.419–2.549)	0.943	1.259 (0.474–3.342)	0.644
Non-amplified	44	1		1	
Implementation of MTC program					
Group 1 (2002–2009)	29	1		1	
Group 2 (2010–2014)	29	0.374 (0.151–0.928)	0.034*	0.591 (0.184–1.901)	0.378

^†^Early-stage.

^‡^Advanced-stage.

*P < 0.05.

CI = confidence interval, EFS = event-free survival, HR = hazard ratio, INSS = International Neuroblastoma Staging System, MTC = multidisciplinary team care, OS = overall survival.

**Figure 3:**
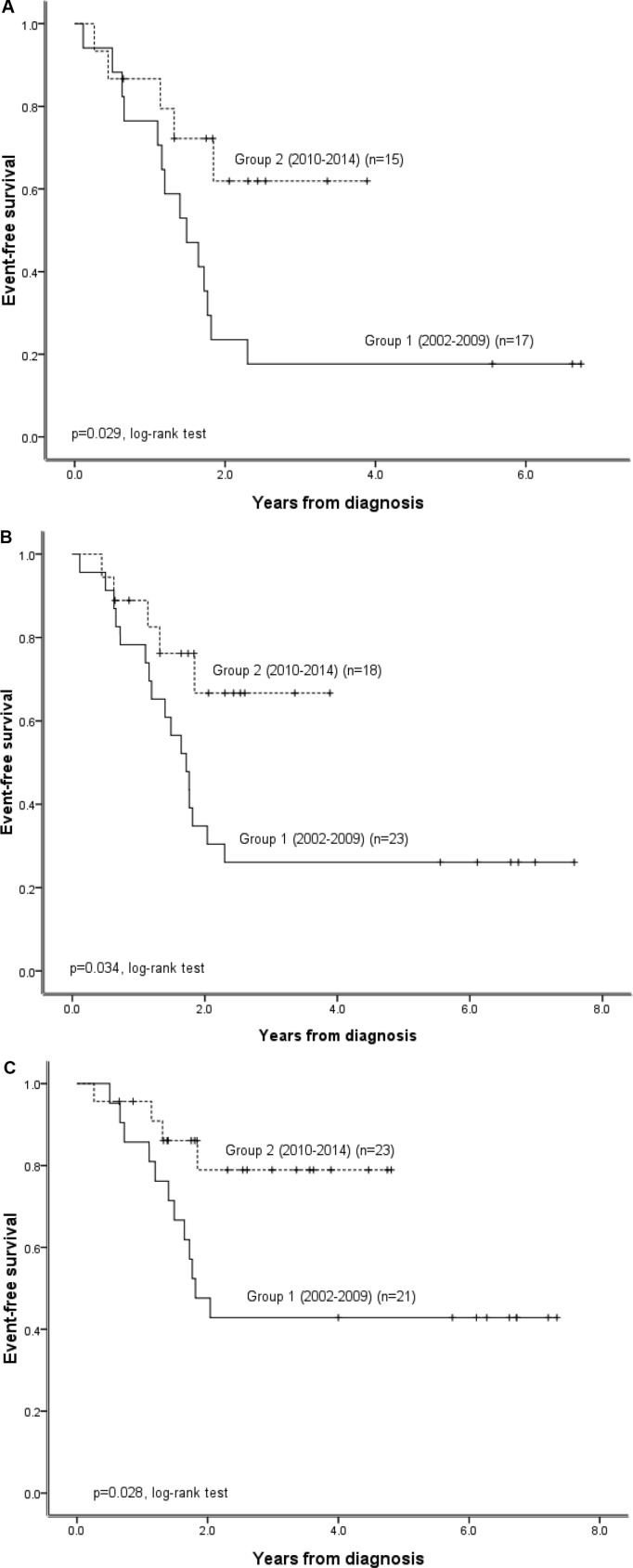
Impact of the implementation of a multidisciplinary team care approach for subgroups of patients with different clinical characteristics treated under the TPOG N2002 protocol (**A**) Probability of EFS in patients with INSS stage 4 disease before (Group 1) and after (Group 2) implementation of the multidisciplinary team care program in 2010. (**B**) Probability of EFS in patients in Group 1 and Group 2 high-risk patients. (**C**) Probability of EFS in non-*MYCN*-amplified Group 1 and Group 2 patients.

We subsequently compared survival outcomes in Group 1 and Group 2 neuroblastoma patients with different clinical characteristics to further understand the prognostic significance of the multidisciplinary team approach. This approach significantly improved EFS, but not OS, in patients with INSS stage 4 disease, those in the high-risk group, and those with non-*MYCN*-amplified tumors under the TPOG N2002 protocol (Figure 3). The multidisciplinary team approach did not affect EFS or OS among non-high-risk group patients (*P* = 0.285, Supplementary Table S1). Univariate analysis of prognostic factors in high-risk group patients revealed that only implementation of the multidisciplinary team approach correlated with better EFS (Supplementary Table S2). In addition, multivariate analysis demonstrated that implementation of the multidisciplinary team approach was the only independent prognostic factor for EFS, but not OS, in high-risk group patients (Supplementary Table S3). Autologous PBSC transplantation neither correlated with better outcomes in univariate analysis nor was identified as an independent prognostic factor in multivariate analysis in high-risk group patients (Supplementary Tables S2 and S3). The gross total tumor resection rate in eligible high-risk group patients tended to be higher in Group 2 (13/16, 81.2%) than in Group 1 (12/22, 54.5%) patients, but this difference did not reach statistical significance (*P* = 0.087, Pearson's chi-square test).

## DISCUSSION

Modern cancer care is complex, and a multidisciplinary team approach may help reduce the potential for poor coordination and miscommunication and improve decision making among the wide range of healthcare professionals involved [5]. In a Canadian review of 30 studies relating to multidisciplinary team care for many types of cancer, Croke *et al*. [13] found that multidisciplinary care usually resulted in changes in diagnosis and management decisions, although there was scant evidence regarding effects on cancer patient survival. In an Australian study, the use of multimodal therapies was associated with the management of Stage IV head and neck cancer patients by a multidisciplinary care team, and it seemed likely that this was also the cause of the improved survival observed in these patients. The treatment outcomes for the neuroblastoma patients involved in this study were comparable to those observed in other studies [3]. Furthermore, our results also indicate that the multidisciplinary team approach improved survival outcomes in high-risk neuroblastoma patients, even when the same multimodal therapy protocol was used. To the best of our knowledge, this is the first report to demonstrate the survival benefits of a multidisciplinary team approach to neuroblastoma care.

Several factors may contribute to the improved survival rates observed in our patients after implementation of the multidisciplinary team care program. The main differences that followed the establishment of the multidisciplinary team approach for neuroblastoma patients were the involvement of a specialized pediatric oncology surgeon and a pediatric oncology nurse practitioner. In a review of a randomized sample of 72 breast cancer teams in England, multidisciplinary teams with higher team workloads and a higher proportion of nurses achieved the best overall clinical performance in newly diagnosed breast cancer patients [14]. Other studies report that high-volume providers have better survival outcomes for complex cancer surgery [15] and that management of cancer patients by surgeons with low workloads decreases OS [16]. During the 8-year period between 2002 and 2009, before the initiation of the multidisciplinary team approach, there were 35 newly diagnosed neuroblastoma patients and 3 pediatric surgeons that performed neuroblastoma surgery in our hospital. Twenty-nine (82.9%) of these patients received the multimodal treatment protocol. One patient (2.8%) died before treatment began, and 5 patients (14.3%) and their families either refused treatment altogether or received treatment under a different protocol. On average, each surgeon performed 1.2 neuroblastoma surgeries per year during this period. There were another 29 newly diagnosed neuroblastoma patients in our hospital during the 5-year period between 2010 and 2014, during which the multidisciplinary team approach was used. All surgical procedures during this period were performed by a specialized pediatric oncology surgeon. None of the patients enrolled after 2010 died before beginning treatment, and all patients received the multimodal treatment protocol. The caseload in our hospital increased after implementation of the multidisciplinary team approach to neuroblastoma care. Furthermore, the nurse-led case management aspect of this approach provided better information for family education, improved patient follow-ups, and assisted patients in accessing the multidisciplinary care team more easily. However, we were unable to demonstrate an association between these characteristics and improvements in outcomes in the present study.

Neuroblastoma patients with stage 4 disease, those in the high-risk group, and those with non *-MYCN*-amplified tumors benefited the most from the multidisciplinary team approach (Figure 3). Although this treatment approach improved only EFS and not OS, autologous bone marrow transplantation has also been reported to improve EFS and not OS in high-risk neuroblastoma patients [17]. However, in that report, Matthay *et al*. also found that autologous bone marrow transplantation appeared to improve OS in patients who survived more than 3 years [17]. The median follow-up time for patients in our study was 3.6 years, and it is possible that the multidisciplinary team approach might improve OS in neuroblastoma patients over longer time periods. We are currently conducting a health-related quality of life investigation using a 36-item short form survey (SF-36) score reported by the parents or care givers of the neuroblastoma patients. This will help us to evaluate changes in patient/parent perception of the treatment and improve the quality of care provided using our multidisciplinary team approach.

Surgical treatment is crucial for the management of neuroblastoma. Gross total tumor resection has a positive prognostic impact in advanced-stage neuroblastoma patients [18, 19], but invading tumors can be heterogeneous and complicated in these patients, making total resection difficult. A multidisciplinary team discussion of patients’ images and the implementation of a preoperative treatment strategy significantly reduce positive circumferential margins in cancer patients [20]. Since 2010, we have regularly reviewed and discussed all neuroblastoma patient images in multidisciplinary team care meetings to define tumor stages, treatment response, and treatment strategies. These practices might improve the accuracy of tumor staging, risk group assignment, surgical procedure planning, and, ultimately, patient clinical outcomes. However, it should be noted that one high-risk Group 1 patient was initially incorrectly assigned to the intermediate-risk group because an epidural metastatic lesion was not noted upon diagnosis, leading to an incorrect classification of stage 3 rather than stage 4 disease. Although this patient was reassigned to the high-risk protocol 3 months later when the lesion was identified in the followed-up image, tumor recurrence occurred one year after diagnosis. All disease stage and risk group assignments were correct for the Group 2 patients.

The limitations of this study, including the non-randomized design, retrospective analysis, and small patient population, should be considered when interpreting the results. These limitations may partially explain why *MYCN* gene amplification was not identified as a statistically significant prognostic factor in the univariate analysis. Additionally, not all neuroblastoma patients with *MYCN*-amplified tumors have poor prognoses; those with early clinical disease stages and hyperdiploid karyotypes were still had favorable outcomes [21]. Because tumor ploidy data was not available for the patients in this study, we were unable to classify *MYCN*-amplified neuroblastoma patients according to the above characteristics.

In conclusion, the treatment results in newly diagnosed neuroblastoma patients in this study were comparable to those obtained in previous studies. Furthermore, we demonstrated that a multidisciplinary team care approach improves survival outcomes in high-risk neuroblastoma patients. However, further investigation is required to evaluate the long-term effects of this approach over longer follow-up periods.

## MATERIALS AND METHODS

### Patient enrollment and study design

Treatment of newly diagnosed neuroblastoma patients under the TPOG N2002 protocol at the National Taiwan University Hospital (Taipei, Taiwan) began in January 2002. Neuroblastoma diagnosis and histological classification were based on the criteria of the International Neuroblastoma Pathology Classification (the Shimada system) [22]. Neuroblastoma staging was performed according to the International Neuroblastoma Staging System [23]. Tumor response was evaluated using the International Neuroblastoma Response Criteria [23]. Clinical data was obtained retrospectively by reviewing patient medical records. The date of last follow-up for patients in this study was December 2014. Patients who either received treatment under a different protocol or refused treatment were excluded from the analysis. The parents or legal guardians of all participants provided informed, written consent. The study was approved by the Institutional Review Board of the National Taiwan University Hospital, Taipei, Taiwan. Research was conducted in accordance with the 1964 Declaration of Helsinki and its later amendments.

### Treatment and chemotherapy regimens

The TPOG N2002 protocol stratified patients into low-, intermediate-, and high-risk groups (Figure 1B). The goal of surgery was to provide diagnostic materials at diagnosis (biopsy), to accurately stage disease by sampling non-adherent lymph nodes, and to attempt maximal safe resection either at diagnosis (excisional biopsy) or after chemotherapy (second-look procedure).

Low-risk neuroblastoma patients with tumor-related respiratory distress, spinal cord/inferior vena cava compression, gastrointestinal/genitourinary obstruction, or systemic coagulopathy who had undergone a partial resection of < 50% of the tumor received 4 cycles of chemotherapy (LR^+^ in Figure 1A; NBL-CT-I, Table 1). Low-risk neuroblastoma patients with localized tumors and without the aforementioned conditions underwent only primary tumor resection (LR in Figure 1A). Intermediate-risk neuroblastoma patients received 8 cycles of chemotherapy (IR in Figure 1A; NBL-CT-I plus NBL-CT-II, Table 1) followed by surgical excision or local radiotherapy of the residual tumor. High-risk neuroblastoma patients received neither the NBL-CT-I or NBL-CT-II regimen. These patients underwent 6 cycles of induction chemotherapy, surgery, consolidation therapy with an autologous PBSC transplantation, and radiotherapy, followed by a 6-month course of oral 13-*cis*-retinoid acid therapy (HR in Figure 1A).

The dosage regimens of the high-risk protocol were based on the N7 [10] and COG-A3973 [12] protocols described previously. Induction chemotherapy cycles 1, 2, 4, and 6 (CDV) consisted of cyclophosphamide (210 mg/m^2^/day or 70 mg/kg/day for patients < 12 kg) on days 1 and 2 and doxorubicin (25 mg/m^2^/day or 0.83 mg/kg/day if < 12 kg) in combination with vincristine (0.67 mg/m^2^/day or 0.022 mg/kg/day, whichever was less; 0.022 mg/kg/day if < 12 kg; 0.017 mg/kg/day for patients < 12 months old) administered as a continuous infusion on days 1 to 3. Induction chemotherapy cycles 3 and 5 (CiE) consisted of cisplatin (50 mg/m^2^/day via a 1-hour infusion or 1.66 mg/kg/day if < 12 kg) on days 1 to 4 and etoposide (200 mg/m^2^/day via a 2-hour infusion or 6.67 mg/kg/day if < 12 kg) on days 1 to 3. PBSCs were harvested after a second course of induction chemotherapy (cyclophosphamide and doxorubicin in combination with vincristine) for patients without disease progression. After 5 cycles of induction chemotherapy, the residual tumor was surgically removed or debulked.

Following 6 cycles of induction chemotherapy, patients exhibiting a complete/very good partial/partial response were encouraged to undergo autologous PBSC transplantation as a consolidation therapy. The myeloablative chemotherapy regimens for transplantation consisted of melphalan (70 mg/m^2^/day or 2.3 mg/kg/day if < 12 kg) administered by bolus infusion on days 1 to 3, and carboplatin (425 mg/m^2^/day or 14.2 mg/kg/day if < 12 kg) combined with etoposide (338 mg/m^2^/day or 11.3 mg/kg/day if < 12 kg) administered by continuous infusion on days 1 to 4. Autologous PBSCs were infused 72 hours after the completion of the above myeloablative consolidation chemotherapy. Patients who were not eligible or who were eligible but decided not to proceed with autologous PBSC transplantation consolidation therapy received 3 cycles of cyclophosphamide (250 mg/m^2^/day or 8.33 mg/kg/day if < 12 kg) in combination with topotecan (0.75 mg/m^2^/day or 0.025 mg/kg/day if < 12 kg) for 5 days as a maintenance therapy. A randomized design was not used for patients who received consolidation (autologous PBSC transplantation) or maintenance therapy because previous reports have already indicated that autologous PBSC transplantation improves disease control rates for high-risk neuroblastoma patients [24, 25].

### The multidisciplinary team care program

In January 2010, we established a multidisciplinary team with expertise in radiology and radiation oncology, pediatric surgery, nuclear medicine, pathology, and pediatric oncology and nursing. The team met once a week in a multidisciplinary conference to review and discuss disease diagnosis, pathology and image findings, staging, and treatment strategies for newly diagnosed neuroblastoma patients, as well as treatment responses in patients receiving therapy. The panel determined disease diagnoses, stages, risk group assignments, and treatment plans for every patient reviewed in the meeting by consensus. One or more physicians together with the case manager then explained the clinical information and discussed the panel's consensus management strategy with the patients’ parents or legal guardian. Case management was led by a pediatric oncology nurse practitioner who provided relevant information for family education and assisted patients in receiving timely access to the multidisciplinary team care program through comprehensive outpatient clinics and regular inpatient visits.

### Statistical analyses

Pearson's chi-square tests were used to assess associations between pairs of categorical variables. Event-free survival (EFS; defined as the time interval from the date of diagnosis to the date of the first event [tumor progression, relapse or death] or last follow-up) and overall survival (OS; defined as the time interval from the date of diagnosis to the date of death or last follow-up) estimates were calculated using the Kaplan-Meier method and compared using the log-rank test. The Cox proportional hazards model was used to identify independent prognostic factors with respect to EFS and OS. All statistical analyses were conducted using Statistical Package for the Social Sciences for Windows, software version 16.0 (SPSS Inc., Chicago, IL, USA). *P*-values < 0.05 were considered statistically significant.

## SUPPLEMENTARY MATERIALS TABLES


