# Supplementary material for: A multidisciplinary team care approach improves outcomes in high-risk pediatric neuroblastoma patients
Source: Oncotarget. 2016 Dec 10;8(3):4360–72. doi: 10.18632/oncotarget.13874 (PMC5354838; doi:10.18632/oncotarget.13874)
Supplement: Supplementary file 1 [file oncotarget-08-4360-s001.pdf]

A multidisciplinary team care approach improves outcomes in high-risk pediatric neuroblastoma patients

Supplementary Materials

Supplementary Table S1: Univariate analysis of prognostic factors in non-high-risk group patients (*n* = 17) with newly diagnosed neuroblastoma

Prognostic factor	Patients (*n* = 17)	3-year EFS, % (SE)	*P*-value	3-year OS, % (SE)
Age at diagnosis			0.009*	
≤ 1.5 years	16	93.8 (6.1)		100
> 1.5 years	1	0		100
INSS stage			0.468	
1, 2, 4S	12	91.7 (8)		100
3, 4	5	80 (17.9)		100
Primary tumor site			0.599	
Adrenal	15	86.7 (8.8)		100
Extra-adrenal	2	100		100
MYCN				
Amplified	0			
Non-amplified	17	88.2 (7.8)		100
Implementation of MTC program			0.285	
Group 1 (2002–2009)	6	100		100
Group 2 (2010–2014)	11	81.8 (11.6)		100

*$P < 0.05$.

EFS = event-free survival, INSS = International Neuroblastoma Staging System, MTC = multidisciplinary team care, OS = overall survival, SE = standard error, TPOG = Taiwan Pediatric Oncology Group.

Supplementary Table S2: Univariate analysis of prognostic factors in high-risk group patients (n = 41) with newly diagnosed neuroblastoma

Prognostic factor	Patients (n = 41)	3-year EFS, % (SE)	P-value	3-year OS, % (SE)	P-value
Age at diagnosis			0.932		0.224
≤ 1.5 years	8	50 (17.7)		72.9 (16.5)	
> 1.5 years	33	36.8 (9.4)		22.7 (9.5)	
INSS stage			0.945		0.856
1, 2, 4S	2	50 (35.4)		50 (35.4)	
3, 4	39	39.8 (8.6)		29.1 (9.8)	
Primary tumor site			0.423		0.106
Adrenal	31	39.2 (9.6)		56 (10.5)	
Extra-adrenal	10	44.4 (16.6)		53.3 (17.3)	
MYCN			0.736		0.677
Amplified	14	39.2 (14)		49 (15)	
Non-amplified	27	40.3 (10.4)		63.1 (10.7)	
Implementation of MTC program			0.034*		0.659
Group 1 (2002–2009)	23	26.1 (9.2)		56.5 (10.3)	
Group 2 (2010–2014)	18	66.7 (12.8)		66.2 (14.5)	
Autologous PBSC transplant			0.593		0.741
Received	27	45 (10.6)		53.8 (11.9)	
Not received	14	32.1 (13)		62.9 (13.3)	

*$P < 0.05$.

EFS = event-free survival, INSS = International Neuroblastoma Staging System, MTC = multidisciplinary team care, OS = overall survival, SE = standard error, TPOG = Taiwan Pediatric Oncology Group.

Supplementary Table S3: Multivariate analysis of prognostic factors in high-risk group patients (*n* = 41) with newly diagnosed neuroblastoma

Prognostic factor	Patients	EFS		OS	
	(*n* = 41)	HR (95% CI)	*P*–value	HR (95% CI)	*P*–value
Age at diagnosis					
≤ 1.5 years	8	0.958 (0.292–3.148)	0.944	0.325 (0.053–1.986)	0.223
> 1.5 years	33	1		1	
INSS stage					
1, 2, 4S[†]	2	1		1	
3, 4[‡]	39	1.713 (0.179–16.358)	0.64	0.685 (0.053–8.807)	0.771
Primary tumor site					
Adrenal	31	2.131 (0.72–6.301)	0.172	2.691 (0.827–8.757)	0.1
Extra-adrenal	10	1		1	
MYCN					
Amplified	14	0.842 (0.316–2.242)	0.73	0.993 (0.362–2.727)	0.99
Non-amplified	27	1		1	
Implementation of MTC program					
Group 1 (2002-2009)	23	1		1	
Group 2 (2010-2014)	18	0.281 (0.095–0.831)	0.022[*]	0.69 (0.204–2.322)	0.55
Autologous PBSC transplant					
Received	27	1.133 (0.443–2.897)	0.795	1.165 (0.427–3.176)	0.766
Not received	14	1		1	

[†]Early-stage.
[‡]Advanced-stage.
[*]$P < 0.05$.
CI = confidence interval, EFS = event-free survival, HR = hazard ratio, INSS = International Neuroblastoma Staging System, MTC = multidisciplinary team care, OS = overall survival.

Supplementary Table S4: Comparing the total dose administered for each drug in NBL-CT-I and NBL-CT-II chemotherapy regimen

	NBL-CT-I	NBL-CT-II
	Cumulative dose (mg/m2)	Cumulative dose (mg/m2)
Carboplatin	1680	1120
Etoposide	1080	720
Cyclophosphamide	2000	3000
Doxorubicin	60	60
